# Discovering the Mechanisms of Oleodaphnone as a Potential HIV Latency-Reversing Agent by Transcriptome Profiling

**DOI:** 10.3390/ijms24087357

**Published:** 2023-04-16

**Authors:** Shifei Li, Xiuyi Wang, Yuqin Yang, Xingkang Wu, Liwei Zhang

**Affiliations:** 1 Key Laboratory of Chemical Biology and Molecular Engineering of Education Ministry, Institute of Molecular Science, Shanxi University, Taiyuan 030006, China; 2 Modern Research Center for Traditional Chinese Medicine, Shanxi University, Taiyuan 030006, China

**Keywords:** *Wikstroemia chamaedaphne*, natural products, daphne H, oleodaphnone, latent HIV activators, transcriptome

## Abstract

Latent HIV is a key factor that makes AIDS difficult to cure. Highly effective and specific latent HIV activators can effectively activate latent HIV, and then combined with antiretroviral therapy to achieve a functional cure of AIDS. Here, four sesquiterpenes (**1**–**4**) including a new one (**1**), five flavonoids (**5**–**9**) including three biflavonoid structures, and two lignans (**10** and **11**) were obtained from the roots of *Wikstroemia chamaedaphne.* Their structures were elucidated through comprehensive spectroscopic analyses. The absolute configuration of **1** was determined by experimental electronic circular dichroism. NH2 cell model was used to test the activity of these 11 compounds in activating latent HIV. Oleodaphnone (**2**) showed the latent HIV activation effect as well as the positive drug prostratin, and the activation effect was time- and concentration-dependent. Based on transcriptome analysis, the underlying mechanism was that oleodaphnone regulated the TNF, C-type lectin receptor, NF-*κ*B, IL-17, MAPK, NOD-like receptor, JAK-Stat, FoxO, and Toll-like receptor signaling pathways. This study provides the basis for the potential development of oleodaphnone as an effective HIV latency-reversing agent.

## 1. Introduction

AIDS is an infectious disease caused by human immunodeficiency virus (HIV) that seriously endangers human health. Combination antiretroviral therapy (cART) can maximize the inhibition of HIV replication, effectively reduce the plasma viral load, prolong the asymptomatic period of infected people, and thus prolong the life of infected people. However, cART cannot completely eradicate HIV-1 in the body, nor can it restore the immune function of infected people. Importantly, the viral load will rebound rapidly after cART is stopped. This is because some HIV-1 will persist stably in some resting CD4^+^ T cells after infection, forming the HIV latency reservoir. Therefore, eliminating the HIV latency reservoir is the key to curing AIDS. At present, the “shock and kill” strategy has become an effective method to eliminate viral reservoirs [1]. Drugs are used to activate the gene transcription (shock) of HIV lurking in cells, and then kill the virus (kill) through the body’s immune system, ART, or other intervention methods to eliminate the latent HIV. Thus, the discovery of efficient and specific HIV latency-reversing agents (LRAs) has become the core of the “shock and kill” strategy.

Natural products have been an important source of drug discovery. At present, many natural activators with latent HIV activation have been found, among which tigliane diterpene prostratin has been under clinical study [2,3,4]. In addition, the daphnane diterpene gnidimacrin not only inhibited HIV replication, but also highly activated latent HIV, which was 2000 times more effective than prostratin [5,6]. Previously a series of tigliane and daphnane diterpenes were isolated from the buds of *Wikstroemia chamaedaphne* by our group [7,8,9]. These diterpenes were shown to activate latent HIV, and transcriptomic analysis showed that they mainly affect RNA synthesis, signal transduction, protein synthesis, and receptor activation [10]. Therefore, in order to explore more novel latent HIV activators, the chemical investigation of the roots *Wikstroemia chamaedaphne* was further studied. In this work, four sesquiterpenes (**1**–**4**) including a new one (**1**), five flavonoids (**5**–**9**) including three biflavonoid structures, and two lignans (**10** and **11**) were obtained and identified (Table 1). Compounds **2**, **6**, and **7** were isolated from this genus for the first time, and compound **5** was isolated from this plant for the first time. NH2 cell model was used to test the activity of these 11 compounds in activating latent HIV, and compound **2** (oleodaphnone) showed a strong effect in activating latent HIV. The effect of oleodaphnone on latent HIV activation showed a time and dose relationship, and the mechanism of oleodaphnone activating latent HIV was further investigated via transcriptomic analysis.

## 2. Results

### 2.1. Phytochemical Investigation of the Roots of Wikstroemia chamaedaphne

In this study, four sesquiterpenes including a new one (**1**, NMR data see Table 2), five flavonoids including three biflavonoid structures, and two lignans were isolated from the roots of *W. chamaedaphne*. The known compounds were identified as oleodaphnone (**2**), 4-epi-15-hydroxyacorenone (**3**), 1*β*-hydroxy-10*β*-H-guaia-4,11-dien-3-one (**4**), genkwanol B (**5**), mesoneochamaejasmin A (**6**), (+)-chamaejasmine (**7**), chrysoeriol (**8**), luteolin (**9**), (+)-pinoresinol (**10**), and 3,4-divanillyltetrahydrofuran (**11**) by comparing their NMR and MS data (Figures S1–S13) with reported values, respectively.

**Figure 1:**
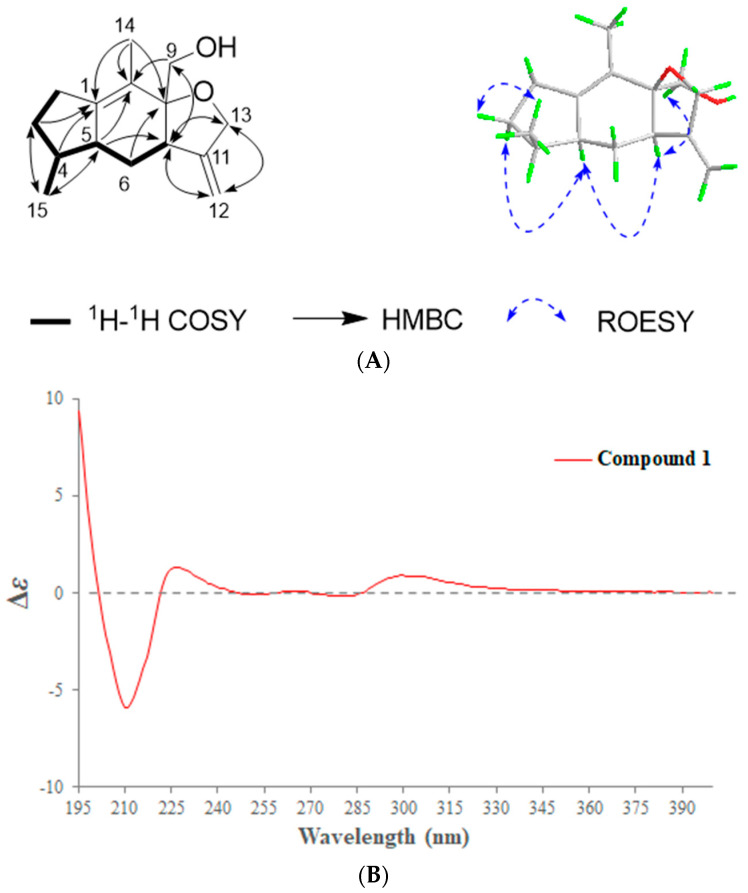
(**A**) The key 2D NMR correlations of compound **1**. (**B**) ECD spectrum of compound **1**.

Compound **1** was isolated as a colorless oil. Its molecular formula was identified as C_15_H_22_O_2_ (five degrees of unsaturation) by its positive-ion HRESIMS (257.1514 [M + Na]^+^, calcd. 257.1512, Figure S1). The ^1^H-NMR spectrum (Figure S2) displayed characteristic signals for one methyl singlet at *δ*_H_ 1.58 (3H, s), one methyl doublet at *δ*_H_ 0.62 (3H, d, *J* = 7.2 Hz), two oxymethylene groups at *δ*_H_ 3.29 (1H, d, *J* = 10.8 Hz, H-9*α*), 3.43 (1H, d, *J* = 10.8 Hz, H-9*β*), 4.21 (1H, d, *J* = 13.2 Hz, H-13*α*), and 4.31 (1H, d, *J* = 13.2 Hz, H-13*β*), and one terminal double bond at *δ*_H_ 4.84 (1H, s, H-12a) and 4.96 (1H, s, H-12b). The ^13^C-NMR and HSQC spectra (Figures S3 and S4) showed that compound **1** had 15 carbon signals, including two groups of double bond signals (*δ*_C_ 153.7, 144.5, 123.7, 104.0), two methyl signals (*δ*_C_ 13.5, 12.3), two sp3 oxymethylene signals (*δ*_C_ 68.9, 63.5), three sp3 methylene signals (*δ*_C_ 31.9, 30.0, 26.6), three sp3 methine signals (*δ*_C_ 45.8, 44.2, 36.0), and one sp3 quaternary carbon signal (*δ*_C_ 86.2). The unsaturation of compound **1** minus the two unsaturations occupied by two groups of double bonds leaves three unsaturations, indicating that compound **1** should be a sesquiterpene with a three-ring system. Further analysis of ^1^H-^1^H COSY and HMBC spectra (Figures S5 and S6) can confirm that compound **1** is a guaiane type sesquiterpene (Figure 1A), and the NMR signal of compound **1** is very similar to that of the known compound daphne A [11]. After detailed comparison with daphne A, it is found that compound **1** had one more methylene signal (*δ*_C_ 26.6) and lacked one oxygenated methine signal (*δ*_C_ 71.1) compared with daphne A. It indicated that compound **1** should be the product of the reduction of 2-hydroxy group in daphne A. This conclusion was further confirmed by ^1^H-^1^H COSY and HMBC data, and this structure was consistent with the molecular weight of compound **1**. Thus, the planar structure of compound **1** was identified as shown in the figure.

The relative configuration of compound **1** was elucidated by the ROESY experiment (Figure S7). The ROESY (Figure 1A, blue arrow) correlations of H-5 to H-2*α* and H-7, H-7 to H_2_-9 indicated that H-2*α*, H-5, H-7, and H_2_-9 were co-facial and assigned as the *α*-configuration. Meanwhile, the ROESY (Figure 1A, blue arrow) correlations of H_3_-15 to H-2*β* indicated that H_3_-15 were assigned as *β*-configuration. Since compound **1** differed from daphne A only in that the hydroxyl group in daphne A was reduced in compound **1**, the absolute configuration of compound **1** can be determined by comparing the CD spectrum with that of daphne A [11]. It can be seen from Figure 1B that the CD spectrum of compound **1** and daphne A had the same Cotton effect, indicating that they had the same configuration. Thus, the absolute configuration of **1** was assigned as 4*S*, 5*S*, 7*R*, 8*S*, and named as daphne H.

### 2.2. Oleodaphnone Is an Efficient HIV LRA

All the isolated compounds were subjected to assay for HIV latency reversal in the HeLa-NH2 cell lines, which is a popular cell model created for studying HIV post-integrative latency [12]. In HeLa-NH2 cells, the reversal response to latent HIV was evaluated by detecting the expression of luciferase. By quantifying luciferase activity in NH2 cells, we were able to generate a functionally relevant drug-response profile defined by the fold changes of luciferase activity.

**Figure 2:**
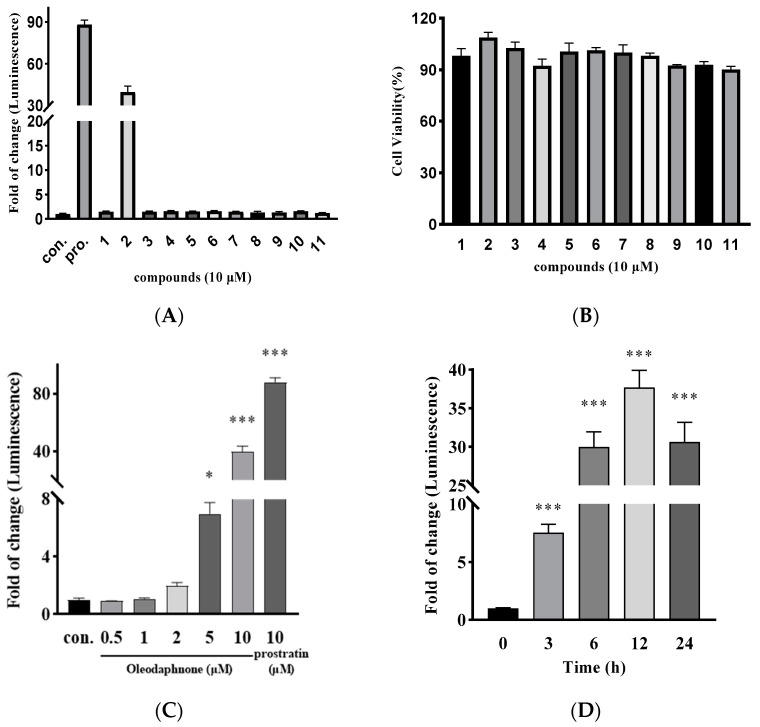
Characterization of oleodaphnone as an HIV latency-reversing agent. (**A**) Histogram illustrating the HIV latency reversal profiles of NH2 cells screened with 11 compounds from *W. chamaedaphne*. NH2 cells were treated with the indicated compounds for 24 h and subjected to HIV latency reversal assays. The color represented the compound. (**B**) Cytotoxicity evaluation of 11 compounds. NH2 cells were treated with 11 compounds at 10 μM for 24 h, and subjected to in vitro cytotoxicity assays. The color represented the compound. Data are presented as mean ±  SEM (*n*  =  3). (**C**) Dose-dependent effects of oleodaphnone on the reactivation of latent HIV. NH2 cells were treated with the indicated concentration of oleodaphnone for 24 h and subjected to HIV latency reversal assays. The color represented the dose. Data are presented as the mean ±  SEM. (*n*  =  3, * *p* < 0.05, *** *p* < 0.001, compared with control). (**D**) Time-dependent effects of oleodaphnone on the reactivation of latent HIV. NH2 cells were treated with 10 μM oleodaphnone for the indicated number of hours and subjected to HIV latency reversal assays. The color represented the time. Data are presented as the mean ±  SEM (*n*  =  3, *** *p* < 0.001, compared with control).

As shown in Figure 2A, all compounds except compound **2** (oleodaphnone) did not show the effect of activating latent HIV at 10 μM. After compound **2** treatment of NH2 cells, the fold change in luciferase activity was 40.25, and the fold changes of luciferase activity for the positive control prostraitin was 88.05. In addition, treatment of NH2 cells with all compounds (10 μM) for 24 h had no effect on cell survival (Figure 2B), and treatment of NH2 cells with different concentrations of compound **2** for 48 h also had no effect on cell survival. To comprehensively assess the HIV latency-reversing activity of oleodaphnone, the NH2 cells line was treated with different concentrations of oleodaphnone, (0.5, 1, 2, 5, and 10 μM) and prostratin (10 μM) for 24 h. Following treatment, the HIV latency-reversing activity was measured, indicating that oleodaphnone reactivated latent HIV in a dose-dependent manner (Figure 2C). Next, we measured the time-dependent reactivation of latent HIV. NH2 cell lines were treated with 10 μM oleodaphnone for 3, 6, 12, and 24 h and assessed for HIV latency-reversing activity. This showed that oleodaphnone reactivated latent HIV in a time-dependent manner (Figure 2D). The maximal HIV latency-reversing activity of oleodaphnone might be achieved with a processing time of approximately 12 h (Figure 2C). Altogether, these data confirmed that oleodaphnone activates latent HIV in a dose- and time-dependent manner, supporting the conclusion that oleodaphnone is an efficient HIV LRA.

As far as we know, oleodaphnone is a guaiane-type sesquiterpenoid originally obtained from *Daphne oleoides* ssp. Oleoides, which did not show any bioactivity or pharmacology effect [13]. This is the first report on the effects of oleodaphnone on HIV. Thus, to extend these findings, the following studies were conducted to understand the mechanism of action of oleodaphnone.

### 2.3. Oleodaphnone Reprograms the Transcriptome of NH2 Cells

**Figure 3:**
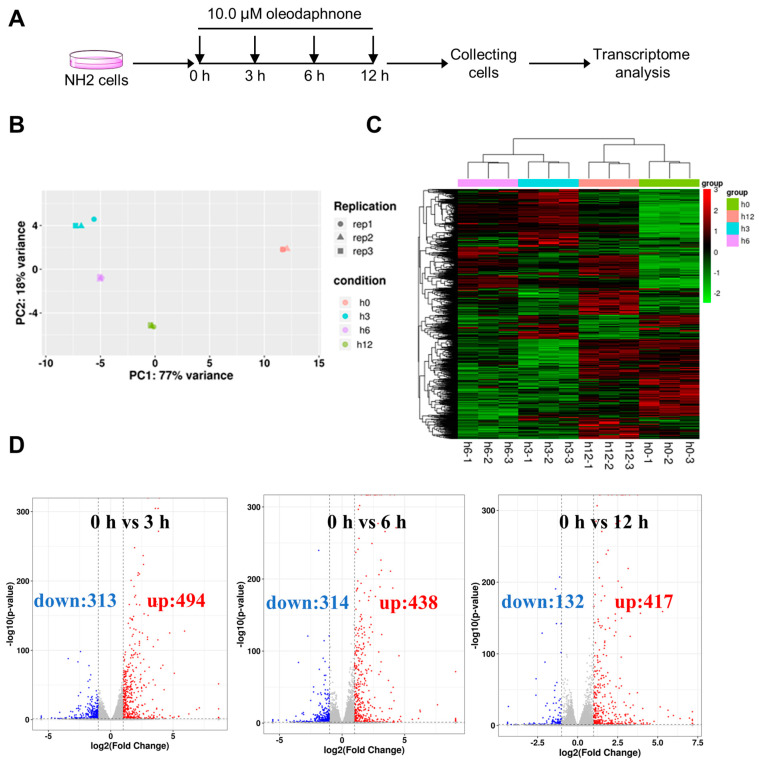
Time course evaluation of the gene transcription profiles of oleodaphnone-treated NH2 cells. (**A**) Diagram of the protocol used to generate samples for transcriptome-wide sequencing. NH2 cell cultures were treated with 10 μM oleodaphnone at the indicated time points and collected at the last time point. (**B**) PCA of 12 independent samples treated under the indicated conditions. The transcript levels of each independent sample were subjected to principal component analysis (PCA) regression of principal components. (**C**) Heatmap showing clustering of differentially expressed genes in NH2 cells treated with oleodaphnone for different times. (**D**) The volcano plot of the mRNA-seq analysis of NH2 cells after oleodaphnone exposure. Red indicates upregulated differentially expressed genes (DEGs), and blue indicates downregulated DEGs.

Given the remarkable potential induction of latent HIV transcription by oleodaphnone, we next sought to dissect the influence of oleodaphnone on the gene transcription profiles of model cells. To address this, transcriptome-wide sequencing (RNA-Seq) was first performed to quantitate gene transcription changes in NH2 cells after treatment with oleodaphnone. NH2 cells were treated with 10 μM oleodaphnone for 0 h, 3 h, 6 h, and 12 h (Figure 3A). Three biological replicates of each treatment were assembled to generate 12 independent samples by collecting cell cultures on different days. The 12 independent samples were grouped into four groups, and the three replicate samples of each group formed a tight cluster in the principal component analysis (PCA) and cluster analysis, showing that samples were available with good reproducibility (Figure 3B,C).

In addition, the clusters of every treatment group were clearly distinguished, indicating that oleodaphnone could change the transcriptome of NH2 cells over the course of treatment (Figure 3B,C). Indeed, a comparison of the gene expression levels among all groups revealed a total of 2108 differentially expressed genes (|log2FoldChange| > 1, *p*-value < 0.05) (Table S1). The 3 h, 6 h and 12 h oleodaphnone treatments upregulated 494, 438, and 417 differentially expressed genes (DEGs), respectively, and downregulated 313, 314, and 132 DEGs (Figure 3D and Table S1). Thus, oleodaphnone induced a time-dependent change in gene expression patterns, suggesting a salient capacity of oleodaphnone to reprogram the transcriptome of NH2 cells.

### 2.4. Identifying Biological Processes and Cell Signaling Responses to Oleodaphnone

**Figure 4:**
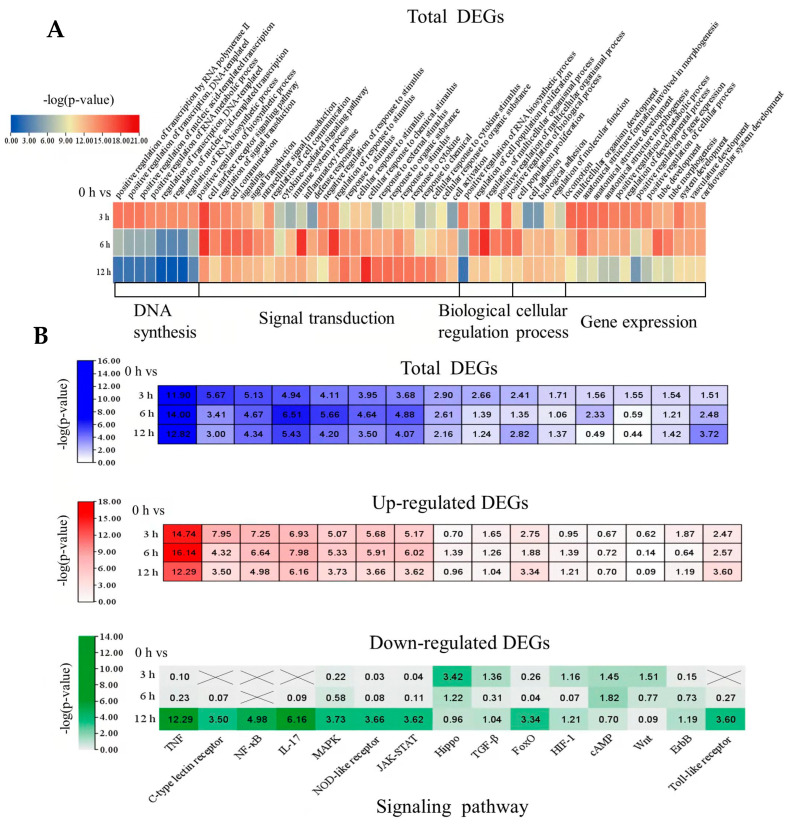
Biological processes and pathways derived from oleodaphnone treatment in NH2 cells. (**A**) Heatmap revealing critical biological processes in oleodaphnone-treated NH2 cells through GO analysis. A negative logarithm (−log10) conversion was performed on the *p*-value obtained in the enrichment test, and the larger the converted value was, the more significant the functional type. (**B**) Heatmap revealing the critical cell signaling pathways in oleodaphnone-treated NH2 cells through KEGG pathway enrichment. A negative logarithm (−log10) conversion was performed on the *p*-value obtained in the enrichment test, and the larger the converted value was, the more significant the functional type.

In order to gain insight into the mechanism of oleodaphnone activation of latent HIV, gene ontology (GO) enrichment analysis was performed on the DEGs in NH2 cells affected by oleodaphnone. A functional enrichment analysis of gene ontology (GO) terms is composed of cellular component, molecular function, and biological process. The biological process reflects the whole biological effect of the cell treated with drugs, so we found that the biological process affected by oleodaphnone mainly included cell surface receptor signaling pathway, cell communication, multicellular organism development, positive regulation of gene expression and defense response, etc. To further analyze the overall change state of the biological processes induced by oleodaphnone at different times, the first 25 GO terms at 3 h, 6 h, and 12 h were selected for heat map analysis (Table S2). As can be seen from Figure 4A, the summarized biological processes in the three time periods were mainly divided into five categories: DNA synthesis, signal transduction, gene expression, biological regulation, and cellular processes. With the extension of treatment time, DNA synthesis and gene expression decreased. With the extension of treatment time, the expression of signal transduction process was up-regulated at 6 h and significantly decreased at 12 h. Among them, the response to stimulus, external stimulus, organic substance, cytokines, and chemicals was enhanced with the extension of time. The regulation and negative regulation of response to stimulus were the strongest at 6 h and the weakest at 12 h. The intracellular response to stimulus, chemicals, cytokines, and organic substance was also enhanced. The process of cell population proliferation and cell adhesion showed the maximum value at 6 h after treatment, and decreased to a certain extent at 12 h. In the biological regulation process, it was clearly observed that the positive regulation of RNA biosynthetic process of NH2 cells treated with oleodaphnone for 6 h showed a significant reduction.

In order to further understand which signaling pathways were significantly affected in oleodaphnone-induced NH2 cells, Kyoto Encyclopedia of Genes and Genomes (KEGG) enrichment analysis was performed to annotate the DEGs. The results showed that a total of 284 signaling pathways were enriched (Table S3). According to *p*-value < 0.05, the top 15 signal pathways with the highest correlation were selected for heat map analysis (Figure 4B). Significantly enriched signaling pathways were found to include TNF, C-type lectin receptor, NF-*κ*B, IL-17, MAPK, NOD-like receptor, JAK-Stat, FoxO, and Toll-like receptor signaling pathways. To further analyze how oleodaphnone significantly altered these signaling pathways at different time periods, the significance levels of up-regulated and down-regulated DEGs at 3 h, 6 h, and 12 h were further analyzed by heat map respectively. The results showed that TNF signaling pathway was most significantly expressed. With the extension of treatment time, C-type lectin receptor and NF-*κ*B signaling pathway were significantly down-regulated, and the most up-regulated DEGs were significantly down-regulated. The significance levels of IL-17, MAPK, and JAK-STAT signaling pathways all showed a peak at 6 h after treatment, and then began to be down-regulated. Hippo and TGF-*β* signaling pathways were down-regulated with the increase in treatment time, most of the down-regulated DEGs in the former were significantly down-regulated, and most of the up-regulated DEGs in the latter were significantly down-regulated. In particular, Wnt signaling pathway was significantly down-regulated in oleodaphnone-induced NH2 cells for 6 h and 12 h. Both up-regulated and down-regulated DEGs showed extremely significant down-regulation.

### 2.5. PPI Network Analysis and Identification of Hub Genes

**Figure 5:**
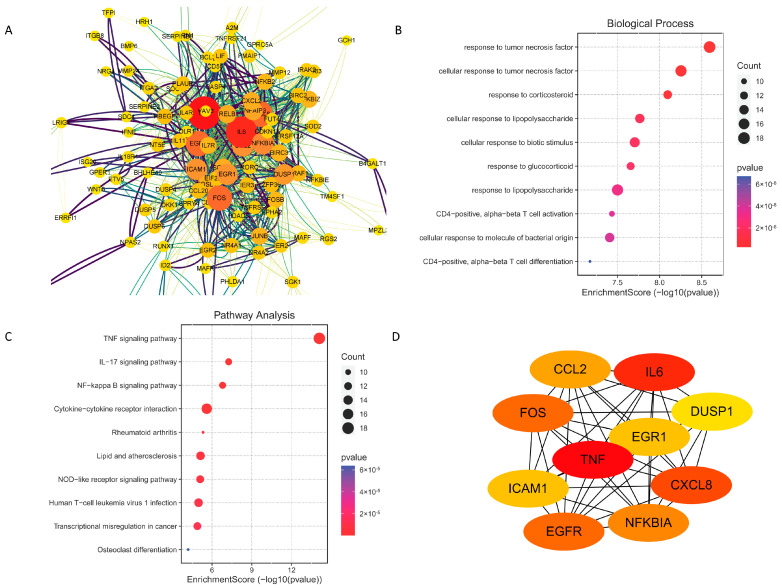
PPI network analysis and identification of Hub Genes. (**A**) PPI network of 194 DEGs. The larger size and darker color represent more interactions of nodes. (**B**) Top 10 significantly enriched GO terms in biological processes (BPs). (**C**) The 20 pathways with the lowest adjusted *p* values. (**D**) PPI network of the hub genes.

The 3 h, 6 h, and 12 h oleodaphnone treatments upregulated 494, 438, and 417 differentially expressed genes (DEGs), respectively, and downregulated 313, 314, and 132 DEGs (Figure 3D and Table S1). Among these results, 194 overlapped DEGs were obtained, including 169 upregulated DEGs and 25 downregulated DEGs. Protein–protein interaction (PPI) network analysis on the overlapped DEGs was performed by using the String database online service platform, and the results including 191 nodes and 599 edges are shown in Figure 5A. Then we performed GO analysis and KEGG pathway analysis on the 194 overlapped DEGs. Finally, 945 BPs, 23 CCs, 63 MFs, and 74 KEGG pathways were obtained (*p* value < 0.05).

GO analysis showed that oleodaphnone might regulate biological processes such as response to tumor necrosis factor, CD4-postive, alpha-beta T cell activation and differentiation, etc (Figure 5B). The KEGG pathway analysis showed that oleodaphnone might regulate TNF signaling pathway, NF-*κ*B signaling pathway, IL-17 signaling pathway, etc (Figure 5C).

In order to obtain the hub genes, we imported the PPI network into the Cytoscape platform, and the Cytohubba plug-in was used for the identification of hub genes. The top 10 nodes generated by degree method were regarded as hub genes, i.e., TNF, IL6, CXCL8, FOS, EGFR, NFKBIA, CCL2, EGR1, ICAM1, and DUSP1. The PPI network of the 10 hub genes had 10 nodes and 44 edges, with an average node degree of 8.8 and *p* value of 6.36 × 10^−13^ (Figure 5D).

### 2.6. Validation on the Changes of TNF and IL-17 Signaling Pathways Induced by Oleodaphnone

**Figure 6:**
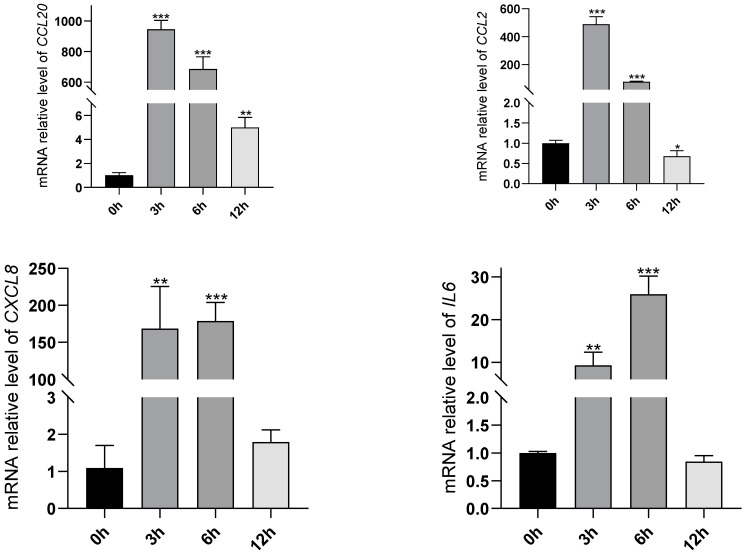
RT-qPCR analysis for *CCL2*, *CCL20*, *CXCL8* and *IL6* genes. (*n*  =  3, * *p* < 0.05, ** *p* < 0.01, *** *p* < 0.001, compared with blank control group).

To evaluate the effect of oleodaphnone on the TNF and IL-17 signaling pathways, we detected mRNA expressions of *CCL2*, *CCL20*, *CXCL8*, and *IL6* genes using RT-qPCR methods. As shown in Figure 6, the expressions of *CCL2*, *CCL20*, *CXCL8*, and *IL6* were significantly increased in the oleodaphnone treated groups compared with blank control group (*p* value < 0.05). At the same time, it was also observed that *CCL2*, *CCL20*, *CXCL8*, and *IL6* genes were highly expressed in oleodaphnone treatment for 3 h. Then, with the extension of treatment time, the expression of *CCL2*, *CCL20*, *CXCL8*, and *IL6* genes began to decline. This trend is consistent with results from transcriptomic.

## 3. Discussion

Since HIV-1 infects host cells, its viral genome can be integrated into the host cell genome to form silent proviruses. These silent proviruses do not transcribe or replicate at very low levels, thus avoiding the attack of the host immune system and antiretroviral and thus remain dormant for a long time [14,15]. However, once the environment changes, such as the withdrawal of antiviral drugs, latent HIV can recover the ability to replicate, leading to rapid deterioration of HIV patients [16,17]. Therefore, efficient elimination of HIV-1 latent reservoir has become the key to overcome the AIDS problem. Currently, LRA has been found to mainly include histone deacetylase inhibitor (HDACi), BRD protein inhibitor, PKC activator, P-TEFb activator, DNA methylase inhibitor, cytokine, etc. Although a few star molecules have entered clinical trials [4,18], the clinical results are not satisfactory, which may be due to the inadequate immune response of the host and the insufficient activation activity of LRA in vivo [19,20]. In addition, LRA can generally cause some toxic side effects, such as over-activation of CD4^+^ T cells and immune disorders, which is also an urgent problem to be solved [21,22]. Therefore, based on the “Shock and Kill” treatment strategy, screening safe and efficient LRA and discovering new activation mechanisms and targets are the foundation and key issues of the current AIDS drug research and development.

In this study, 11 compounds isolated from the stem of *Wikstroemia chamaedaphne* were tested for the activation of latent HIV for the first time, and one guaiane type sesquiterpene oleodaphnone showed strong activation of latent HIV. Further study showed that oleodaphnone activated latent HIV in a time- and concentration-dependent manner, indicating that oleodaphnone was a potential LRA. The transcriptomic profile also showed that the biological processes of NH2 cells regulated by oleodaphnone were significantly time dependent. In particular, two biological processes, DNA synthesis and gene expression, which were significantly affected by oleodaphnone after 3 h, returned to normal after 12 h. Similarly, the signal pathways regulated by oleodaphnone were evident after 3 h of treatment, but these pathways began to be reversed after 6 h and 12 h. It can be seen that the time dependence of the biological processes and signaling pathways affected by oleodaphnone was just consistent with the effect of oleodaphnone on the activation of latent HIV, and the best time for oleodaphnone to activate latent HIV was 3 h. Since HIV is main latent in the CD4^+^ T cell genome, it took time for oleodaphnone to activate latent HIV expression before it reached the genome. In addition, this activation process was a drug metabolism process. The time-dependent results of oleodaphnone activation of latent HIV are different from those of wikstroelide E [10]. The biological processes and signaling pathways affected by oleodaphnone and wikstroelide E are also quite different. Although oleodaphnone also affected signaling pathways such as NF-*κ*B, JAK-STAT, MAPK, and HIPPO, which had been shown to be effective in activating latent HIV, oleodaphnone displayed the most significant effects on TNF signaling, and C-type lectin receptor signaling. Many studies have shown that TNF plays an important role in HIV infection and progression; for example, demonstrating that hepatitis C virus core protein can enhance HIV-1 replication and activate latent HIV expression in human macrophages by upregulating TNF-*α* [23,24]. C-type lectin expressed on dendritic cells (DCs), can sequester HIV virus in multi-vesicular bodies [25]. A specific C-type lectin receptor expressing in Langerhans cells, named langerin, is involved in HIV capture and destruction [26]. HIV is an extremely harmful virus, HIV mainly infects a variety of immune cells with CD4^+^ T and chemokine receptors CXCR4/CCR5 on their surface, making them in a latent infection state [27]. Therefore, although the extent to which the compounds activate latent HIV activity is different, the induced signaling pathways may have similarities and particularities. This may be related to the target of latent HIV activator. PKC may be the direct target of diterpene activation of latent HIV like wikstroelide E, but the target of oleodaphnone may be more related to TNF, IL6, CXCL8, FOS, EGFR, NFKBIA, CCL2, EGR1, ICAM1, and DUSP1.

As far as we know, oleodaphnone was first isolated from *Daphne oleoides* ssp. Oleoides [13]. Oleodaphnone showed neither cytotoxic effect against MKN-45, SKOV3, and Du145 cell lines at 20 μM nor inhibitory effects on ferroptosis in HT-22 cells at 40 μM [28,29]. In addition, then no biological activity was reported about oleodaphnone. This is the first report of the activation on latent HIV of oleodaphnone, and also the first report of the activation on latent HIV by this sesquiterpene. However, compound **3**, also guaiane type sesquiterpene, did not show the effect of activating latent HIV. The difference between oleodaphnone and compound **3** is that oleodaphnone has an extra pair of *α*,*β*-unsaturated ketone. The *α*,*β*-unsaturated ketone are known to form stable covalent bonds with cysteine residues via Michael addition. We speculate that oleodaphnone may form complexes with some key targets regulating the latent HIV and inhibit the function of these targets, thus playing a role in activating latent HIV. Therefore, exploring the target of oleodaphnone to activate latent HIV may provide a basis for the discovery of novel biological activities of this sesquiterpene.

## 4. Materials and Methods

### 4.1. General Experimental Procedure

The ECD spectra were obtained with a BioLogic MOS-450 spectropolarimeter (BioLogic Co., Ltd., Grenoble, French). The 1D and 2D spectra were recorded on a Bruker-ARX-600 spectrometer (600 and 150 MHz, Karlsruhe, Germany): *δ* in ppm, *J* in Hz. High resolution electrospray ionization mass measurements (HRESIMS) were recorded in positive ion mode on a Micro TOF-Q (quadrupole-Time of Flight) instrument with a Bruker ESI source. Semipreparative HPLC was performed with an Agilent 1200 series (California, Agilent Technologies Co., Ltd., USA) with UV detection, using a C18 Agilent column (9.4 × 250 mm, 5 μm; Agilent) at a flow rate of 3.0 mL/min. Column chromatography was performed on silica gel (10–40 μm, Qingdao Marine Chemical Factory, Qingdao, China) and Sephadex LH-20 (40–70 μm, Amersham Pharmacia Biotech AB, Uppsala, Sweden). MPLC was performed on Büchi Sepacore System (Büchi Labortechnik AG, Flawil, Switzerland), and columns packed with ODS C18 (40–75 µm, Fuji Silysia Chemical Ltd., Mizuho-Tori, Japan).

### 4.2. Plant Material

The roots of *Wikstroemia chamaedaphne* Meisn. were collected from Jiangxian County (latitude 35°52′ N; longitude 111°49′ E), Shanxi Province, People’s Republic of China, in July 2018, and identified by Prof. Feng Zhang at Institute of Loess Plateau, Shanxi University, China. The voucher specimen (No. 20180702C) was deposited in the Key Laboratory of Chemical Biology and Molecular Engineering of Education Ministry, Institute of Molecular Science, Shanxi University, China.

### 4.3. Extraction and Isolation

The air-dried root of *W. chamaedaphne* (5 Kg) was extracted with ethanol (50 L) under reflux two times, 2 h for each time. The combined extracts were concentrated to yield a dry residue (420 g). This residue was divided into six fractions (A-F) by silica gel column chromatography. Fraction C (50.0 g) was chromatographed on C-18 eluted with MeOH/H_2_O (1:4–5:0) to afford subfractions C1-C7. Subfraction C6 (1.7 g) was subjected to Sephadex LH-20 eluted with MeOH/H_2_O (9:1, *v/v*) to yield C6a and C6f. C6d was followed by purification over semipreparative HPLC (85% MeOH in H_2_O) to yield compounds **1** (1.2 mg) and **2** (8.6 mg). Subfraction C5 (29.5 g) was subjected to gel chromatography again, and divided into three subfractions (C5a-C5c) by the mobile phase was CHCl_3_/MeOH. Subfraction C5c was followed by purification over semipreparative HPLC to yield compounds **3** (5.2 mg), **4** (23.2 mg), **10** (9.3 mg), and **11** (35.8 mg). Subfraction C5a (2.6 g) was eluted by gel Sephadex LH-20 (chloroform/methanol 1:1) and recrystallized to obtain compound **8** (4.3 mg). Subfraction C4 (32.3 g) was subjected to column chromatography eluted with CHCl_3_ containing an increasing amount of MeOH to afford C4a-C4d. C4c (32.3 g) was subjected to Sephadex LH-20 (eluted with MeOH), followed by purification over semipreparative HPLC to yield compounds **5** (9.5 mg), **6** (38.7 mg), and 7 (20.3 mg). After the same column chromatography, compound **9** (8.8 mg) was isolated from component C4d.

### 4.4. Cell

NH2 cells were generated with pcDNA3-Tat-HA by transfection of NH1 cells containing an integrated HIV-1 LTR-luciferase reporter, and then, they were selected with G418 to obtain a stable clone [30]. The production of Tat-HA in the G418-resistant clone NH2 was confirmed by western blotting [31]. NH2 cell lines were cultured in DMEM complete medium. All complete medium was supplemented with 10% fetal bovine serum (Sijiqing; Zhejiang Tianhang Biotechnology Co., Ltd., Hangzhou, China), 50 μg/mL penicillin, and 50 μg/mL streptomycin.

### 4.5. HIV Latency-Reversing Assays

Luciferin was used as a substrate to detect luciferase activity. During incubation, the HIV pro-viral gene was silenced, so luciferase was not expressed in the cell. After being activated, the HIV pro-viral gene begins to be expressed, and luciferase is also expressed in cells. The expression of luciferase indicated that the HIV provirus in NH2 cells was activated for transcription. The cells were treated with compounds and then subjected to a luciferase assay with a Luciferase Reporter Gene Assay Kit (RG005, Beyotime Biotechnology, Shanghai, China). The ability of the drug (compounds) to activate latent HIV was indicated by the fold change in luciferase activity and calculated by the following formulas.

Fold change (luciferase activity) = L_drug_/L_DMSO_, where drug indicates the drug-treated groups and DMSO indicates the DMSO-treated groups as drug-untreated groups.

### 4.6. Transcriptome Analysis

Oleodaphnone (10 μM) was added to the cells incubated for 0 h, 3 h, 6 h, and 12 h, and three parallel wells were established in each group. The cells were collected and transferred to a 15 mL centrifuge tube at 500× *g* for 5 min, and the supernatant was removed. TRIzol (1 mL) was added to every 5 × 10^6^ cells. RNA was extracted with TRIzol and 75% ethanol. After extracting RNA, total RNA quality inspection, mRNA purification, mRNA fragmentation, cDNA synthesis, and library quality inspection were performed. Based on the Illumina HiSeq sequencing platform, paired-end sequencing was performed to obtain the raw data in FASTQ format (Raw Data). Then, Sanger quality value evaluation was performed on the off-machine data, with statistically filtering and sorting of the data. FastQC was used to perform quality inspection again, focusing on four indicators: GC content distribution, base content distribution, single base quality, and sequence base quality. Then, expression analysis was performed to evaluate the data quality. Differentially expressed genes were screened. Through the comparison of gene expression (RPKM), DESeq was used to analyze the differential genes. The screening conditions were as follows: expression fold difference|log2fold change| > 1, significance *p* value < 0.05.

### 4.7. Bioinformatics Analysis

GO-Term Finder was used to identify GO terms that annotate a list of enriched genes with a significant *p*-value < 0.05 [32]. The KEGG is a collection of databases dealing with genomes, biological pathways, diseases, drugs, and chemical substances (https://www.kegg.jp/ (accessed on 1 February 2023)). In-house scripts were used to find KEGG pathways enriched in DEGs. All unigene sequences were also annotated using BLAST against the nr, Clusters of Orthologous Genes (COG), Swiss-Prot, KEGG, and GO databases. PPI network analysis on the DEGs was performed by using the String database online service. Visualization of PPI network was performed on software Cytoscape and the Cytohubba plug-in was used for the identification of hub genes.

### 4.8. RT-qPCR Analysis

Total RNA was extracted with an RNA Extraction Kit (Takara), and reverse transcribed with RT Master Mix (Takara) according to the manufacturer’s protocol. Then, the generated cDNA was subjected to quantitative RT-PCR analysis, with the use of TB Green^®^ Premix (Takara) and a QuantStudio 96 RT-PCR system (Thermo). Actin protein β-actin was used as an internal control. The primer sequences were shown in Table 3.

### 4.9. Statistical Analysis

Data are representative of three independent experiments, and error bars represent the standard deviation. Two treatment groups were compared by the two-tailed unpaired Student’s t test using Microsoft Excel and Prism 7.0 (GraphPad). Statistical significance is indicated at * *p* < 0.05, ** *p* < 0.01 or *** *p* < 0.001.

## 5. Conclusions

In conclusion, 11 compounds including a new guaiane type sesquiterpene were obtained from the roots of *Wikstroemia chamaedaphne.* The structure of the new sesquiterpene along with the absolute configuration was elucidated by spectroscopic analysis. All the 11 compounds were tested to activate latent HIV, and only sesquiterpene oleodaphnone was shown to be effective in activating latent HIV. Oleodaphnone activated latent HIV in a time- and concentration-dependent manner, demonstrating that oleodaphnone is a potent HIV LRA for HIV therapy. Mechanistically, transcriptome analysis indicated that oleodaphnone regulated the TNF, C-type lectin receptor, NF-*κ*B, IL-17, MAPK, NOD-like receptor, JAK-Stat, FoxO, and Toll-like receptor signaling pathways. Indeed, PPI analysis revealed that TNF, IL6, CXCL8, FOS, EGFR, NFKBIA, CCL2, EGR1, ICAM1, and DUSP1 might be the targets of oleodaphnone to activate latent HIV.

## Figures and Tables

**Table 1:** Chemical constituents from the roots of *Wikstroemia chamaedaphne*.

Sesquiterpene	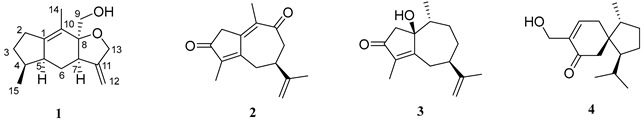
Flavonoid	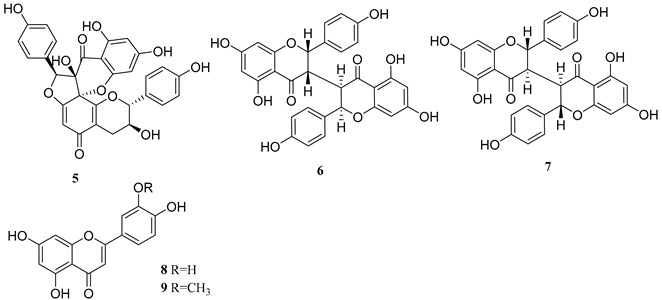
Lignan	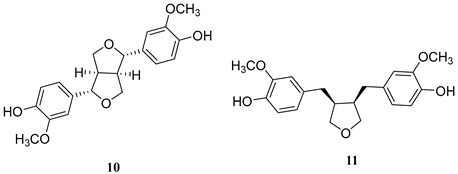

**Table 2:** NMR data of compounds **1**–**4**.

NO.	1 (CD_3_OD)		2 (CD_3_OD)		3 (CDCl_3_)		4 (CD_3_OD)	
	^1^H	^13^C		^13^C	^1^H	^13^C	^1^H	^13^C
1		144.5		144.8		83.4	1.32, overlap	56.9
2	2.12, overlap	26.6	3.18, q (21.0)	40.6	2.61, d (18.0)	52.3	1.67, overlap	25.8
					2.43, d (18.0)		1.32, overlap	
3	1.65, overlap	31.9		203.1		207.8	1.67, overlap	29.8
	1.41, dd (12.6, 7.8)						1.32, overlap	
4	2.12, overlap	36.0		145.1		139.1	1.67, overlap	46.4
5	2.21, overlap	44.2		163.9		174.6		48.2
6	1.61, overlap	30.0	3.05, d (16.8)	35.3	2.74, d (18.6)	36.9	2.76, d (16.8)	49.5
	1.01, d (12.6)		2.91, overlap		2.62, d (18.0)		2.24, d (16.8)	
7	2.70, dd (13.2, 6.0)	45.8	2.76, m	39.3	2.27, overlap	44.0		201.5
8		86.2	2.91, overlap	49.6	1.81, m	31.3		138.0
					1.54, m			
9	3.43, d (10.8)	63.5		201.5	1.65, m	31.9	6.84, m	145.9
	3.29, d (10.8)				1.54, m			
10		123.7		131.6	2.27, overlap	41.2	2.36, d (19.8)	25.4
							2.13, d (19.8)	
11		153.7		147.9		152.5	1.67, overlap	29.4
12	4.96, s	104.0	4.83, d (9.0)	110.3	4.78, s	109.4	0.78, t (9.6)	24.2
	4.84, s				4.71, s			
13	4.31, d (13.2)	68.9	1.82, s	19.4	1.79, s	20.5	0.95, t (9.6)	21.6
	4.21, d (13.2)							
14	1.58, s	12.3	1.98, s	16.3	1.68, s	14.6	0.87, m	17.3
15	0.62, d (7.2)	13.5	1.87, s	7.8	0.77, d (7.2)	7.8	4.24, s	62.2

**Table 3:** The sequences of the primers.

Primer	Forward Primer (5′ to 3′)	Reverse Primer (5′to 3′)
*CCL2*	AGAATCACCAGCAGCAAGTGTCC	TCCTGAACCCACTTCTGCTTGG
*CCL20*	TGACTGCTGTCTTGGATACACAGA	TGATAGCATTGATGTCACAGCCT
*CXCL8*	GTCCTTGTTCCACTGTGCCT	GCTTCCACATGTCCTCACAA
*IL6*	AGACAGCCACTCACCTCTTCAG	TTCTGCCAGTGCCTCTTTGCTG
*β-actin*	AAGGATTCCTATGTGGGCGAC	CGTACAGGGATAGCACAGCC

## Data Availability

The raw data supporting the conclusions of this manuscript will be made available by the authors, without reservation, to any qualified researcher.

## Supplementary Materials

The supporting information can be downloaded at: https://www.mdpi.com/article/10.3390/ijms24087357/s1.

## Abbreviations

HIV	Human immunodeficiency virus
AIDS	Acquired immunodeficiency syndrome
cART	Combination antiretroviral therapy
LRAs	Latency-reversing agents
NMR	Nuclear magnetic resonance
HRESIMS	High resolution electrospray ionization mass spectroscopy
HSQC	Heteronuclear signal quantum coherence
^1^H-^1^H COSY	^1^H-^1^H Correlation spectroscopy
HMBC	Heteronuclear multiple bond correlation
ROESY	Rotating-Frame Overhauser Enhancement and Exchange Spectroscopy
ECD	Electronic Circular Dichroism
PCA	Principal component analysis
DEGs	Different expression genes
GO	Gene ontology
KEGG	Kyoto Encyclopedia of Genes and Genomes
PPI	Protein–protein interaction
BP	Biological process
CC	Cellular component
MF	Molecular Function
PKC	protein kinase C
P-TEFb	Positive transcription elongation factor b
HDACi	Histone deacetylase inhibitor
HPLC	High Performance Liquid Chromatography
LH-20	Sephadex LH-20
s	Singlet (Spectroscopic)
d	Doublet (Spectroscopic)
*J*	Coupling constant (in NMR)
*δ*	Chemical shift in parts per million downfield from TMS
2D NMR	Two dimensional NMR

## References

[1] Rasmussen T.A., Tolstrup M., Sgaard O.S. (2015). Reversal of latency as part of a cure for HIV-1. Trends Microbiol..

[2] Wang X.Y., Jiao Y.Y., Zhang L.W., Li S.F. (2021). Research progress on natural product human immunodeficiency virus-1 (HIV-1) latency reactivation agents. Zhongcaoyao.

[3] Kulkosky J., Culnan D.M., Roman J., Dornadula G., Schnell M., Boyd M.R., Pomerantz R.J. (2001). Prostratin: Activation of latent HIV-1 expression suggests a potential inductive adjuvant therapy for HAART. Blood.

[4] Wender P.A., Kee J.M., Warrington J.M. (2008). Practical synthesis of prostratin, DPP, and their analogs, adjuvant leads against latent HIV. Science.

[5] Huang L., Ho P., Yu J., Zhu L., Lee K.H., Chen C.H. (2011). Picomolar dichotomous activity of gnidimacrin against HIV-1. PLoS ONE.

[6] Lai W., Huang L., Zhu L., Ferrari G., Chan C., Li W., Lee K.H., Chen C.H. (2015). Gnidimacrin, a potent anti-HIV diterpene, can eliminate latent HIV-1 ex vivo by activation of protein kinase C β. J. Med. Chem..

[7] Li S.F., Jiao Y.Y., Zhang Z.Q., Chao J.B., Jia J., Shi X.L., Zhang L.W. (2018). Diterpenes from buds of *Wikstroemia chamaedaphne* showing anti-hepatitis B virus activities. Phytochemistry.

[8] Zhang Z., Li S.F., Zhang L.W., Chao J.B. (2017). Chemical constituents from flowers of *Wikstroemia chamaedaphne* and their anti-hepatitis B virus activity. Zhongcaoyao.

[9] Li S.F., Wang X.Y., Li G.L., Jiao Y.Y., Wang W.H., Wu X.K., Zhang L.W. (2022). Potential HIV latency-reversing agents with STAT1-activating activity from the leaves of *Wikstroemia chamaedaphne*. Phytochemistry.

[10] Li S.F., Liang X., Wu X.K., Gao X., Zhang L.W. (2021). Discovering the mechanisms of wikstroelide E as a potential HIV-latency-reversing agent by transcriptome profiling. J. Nat. Prod..

[11] Wang J., Liu Q.B., Hou Z.L., Shi S.C., Ren H., Yao G.D., Lin B., Huang X.X., Song S.J. (2020). Discovery of guaiane-type sesquiterpenoids from the roots of *Daphne genkwa* with neuroprotective effects. Bioorg. Chem..

[12] Haas B.J., Papanicolaou A., Yassour M., Grabherr M., Blood P.D., Bowden J., Couger M.B., Eccles D., Li B., Lieber M. (2013). De novo transcript sequence reconstruction from RNA-seq using the Trinity platform for reference generation and analysis. Nat. Protoc..

[13] Taninaka H., Takaishi Y., Honda G., Imakura Y., Sezik E., Yesilada E. (1999). Terpenoids and aromatic compounds from *Daphne oleoides* ssp. *oleoides*. Phytochemistry.

[14] Kimata J.T., Rice A.P., Wang J. (2016). Challenges and strategies for the eradication of the HIV reservoir. Curr. Opin. Immunol..

[15] Van Lint C., Bouchat S., Marcello A. (2013). HIV-1 transcription and latency: An update. Retrovirology.

[16] Bandera A., Gori A., Clerici M., Sironi M. (2019). Phylogenies in ART: HIV reservoirs, HIV latency and drug resistance. Curr. Opin. Pharmacol..

[17] Dahabieh M.S., Battivelli E., Verdin E. (2015). Understanding HIV latency: The road to an HIV cure. Annu. Rev. Med..

[18] Kalvatchev Z., Walder R., Garzaro D. (1997). Different effects of phorbol ester derivates on human immunodeficiency virus 1 replication in lymphocytic and monocytic human cells. Acta Virol..

[19] De la Torre-Tarazona H.E., Jiménez R., Bueno P. (2020). 4-Deoxyphorbol inhibits HIV-1 infection in synergism with antiretroviral drugs and reactivates viral reservoirs through PKC/MEK activation synergizing with vorinostat. Biochem. Pharmacol..

[20] Shang H.T., Ding J.W., Yu S.Y., Wu T., Zhang Q.L., Liang F.J. (2015). Progress and challenges in the use of latent HIV-1 reactivating agents. Acta Pharmacol. Sin..

[21] Este J.A., Cihlar T. (2010). Current status and challenges of antiretroviral research and therapy. Antivir. Res..

[22] Eisele E., Siliciano R.F. (2012). Redefining the viral reservoirs that prevent HIV-1 eradication. Immunity.

[23] Swaminathan G., Pascual D., Rival G., Perales-Linares R., Martin-Garcia J., Navas-Martin S. (2014). Hepatitis C virus core protein enhances HIV-1 replication in human macrophages through TLR2, JNK, and MEK1/2-dependent upregulation of TNF-α and IL-6. FEBS Lett..

[24] Lazdins J.K., Grell M., Walker M.R., Woods-Cook K., Scheurich P., Pfizenmaier K. (1997). Membrane tumor necrosis factor (TNF) induced cooperative signaling of TNFR60 and TNFR80 favors induction of cell death rather than virus production in HIV-infected T cells. J. Exp. Med..

[25] Hodges A., Sharrocks K., Edelmann M., Baban D., Moris A., Schwartz O., Drakesmith H., Davies K., Kessler B., McMichael A. (2007). Activation of the lectin DC-SIGN induces an immature dendritic cell phenotype triggering Rho-GTPase activity required for HIV-1 replication. Nat. Immunol..

[26] Mayr L., Su B., Moog C. (2017). Langerhans Cells: The ‘Yin and Yang’ of HIV Restriction and Transmission. Trends Microbiol..

[27] Castedo M., Perfettini J.L., Andreau K., Roumier T., Piacentini M., Kroemer G. (2003). Mitochondrial apoptosis induced by the HIV-1 envelope. Ann. N. Y. Acad. Sci..

[28] Yang F., Jiang X., Cao L., Gu Q., Teng X., He L. (2022). Diverse sesquiterpenoids from the roots of *Croton crassifolius* and their inhibitory effects on ferroptosis. Chem. Biodivers..

[29] Pan J., Su J.C., Liu Y.H., Deng B., Hu Z.F., Wu J.L., Xia R.F., Chen C., He Q., Chen J.C. (2021). Stelleranoids A-M, guaiane-type sesquiterpenoids based on [5,7] bicyclic system from *Stellera chamaejasme* and their cytotoxic activity. Bioorg. Chem..

[30] Yang Z., Zhu Q., Luo K., Zhou Q. (2001). The 7SK small nuclear RNA inhibits the CDK9/cyclin T1 kinase to control transcription. Nature.

[31] Li Z., Guo J., Wu Y., Zhou Q. (2013). The BET bromodomain inhibitor JQ1 activates HIV latency through antagonizing Brd4 inhibition of Tat-transactivation. Nucleic Acids Res..

[32] Boyle E.I., Weng S., Gollub J., Jin H., Botstein D., Cherry J.M., Sherlock G. (2004). GO::TermFinder—Open source software for accessing Gene Ontology information and finding significantly enriched Gene Ontology terms associated with a list of genes. Bioinformatics.

