# Supplementary material for: Discovering the Mechanisms of Oleodaphnone as a Potential HIV Latency-Reversing Agent by Transcriptome Profiling
Source: Int J Mol Sci. 2023 Apr 16;24(8):7357. doi: 10.3390/ijms24087357 (PMC10138910; doi:10.3390/ijms24087357)

Supplementary Materials for

Discovering the mechanisms of oleodaphnone as a potential HIV latency-reversing agent by transcriptome profiling

Content

S1. Supporting methods: In vitro cytotoxicity assay.

Figure S1. HRESIMS spectrum of **1**.

Figure S2. ^{1}H-NMR spectrum of **1**.

Figure S3. ^{13}C-NMR spectrum of **1**.

Figure S4. HSQC spectrum of **1**.

Figure S5. ^{1}H-^{1}H COSY spectrum of **1**.

Figure S6. HMBC spectrum of **1**.

Figure S7. ROESY spectrum of **1**.

Figure S8. ^{1}H-NMR spectrum of **2**.

Figure S9. ^{13}C-NMR spectrum of **2**.

Figure S10. ^{1}H-NMR spectrum of **3**.

Figure S11. ^{13}C-NMR spectrum of **3**.

Figure S12. ^{1}H-NMR spectrum of **4**.

Figure S13. ^{13}C-NMR spectrum of **4**.

S1. Supporting methods: In vitro cytotoxicity assay

The Cell Counting Kit-8 (CCK-8) (Dojindo, Kumamoto, Japan) was used to measure the in vitro cytotoxicity of the compound. Briefy, approximately 4×10^4 cells per well were treated with Wikstroelide E or prostratin for 24 h, and then 10 μl of CCK-8 solution was added to each well of the 96-well culture plates. Afer 4 h of incubation at 37°C, the absorbance at 450 nm was measured using a microplate reader. The 50% cytotoxic concentration (CC50) was calculated by nonlinear regression analysis using GraphPad Prism 5 sofware (GraphPad, SanDiego, CA).

Figure S1. HRESIMS spectrum of **1**.

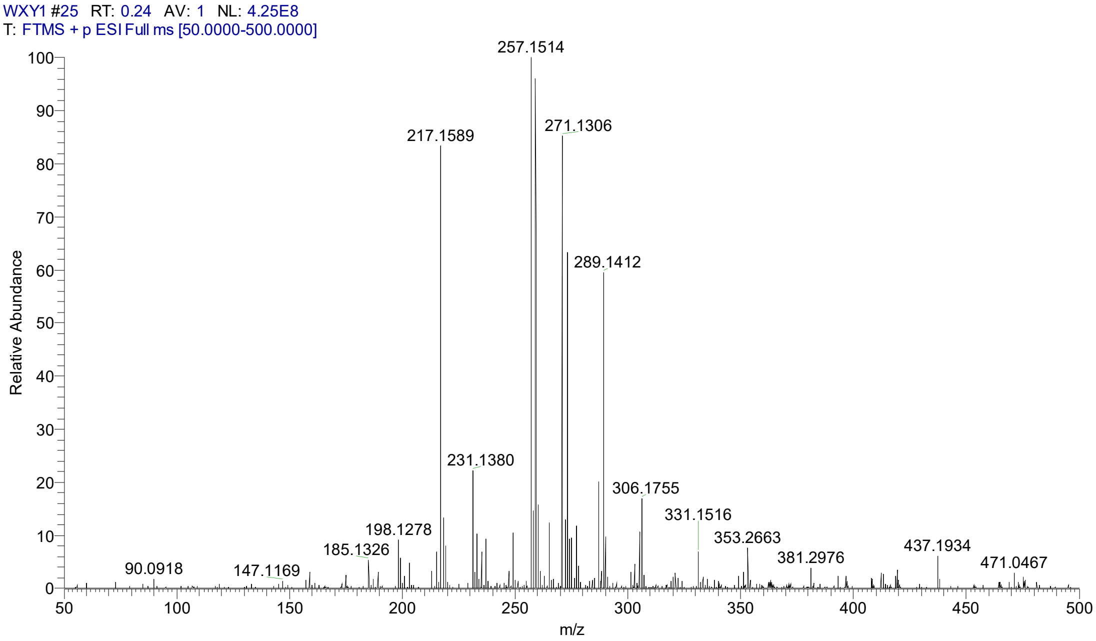

Figure S2. ^{1}H-NMR spectrum of **1**.

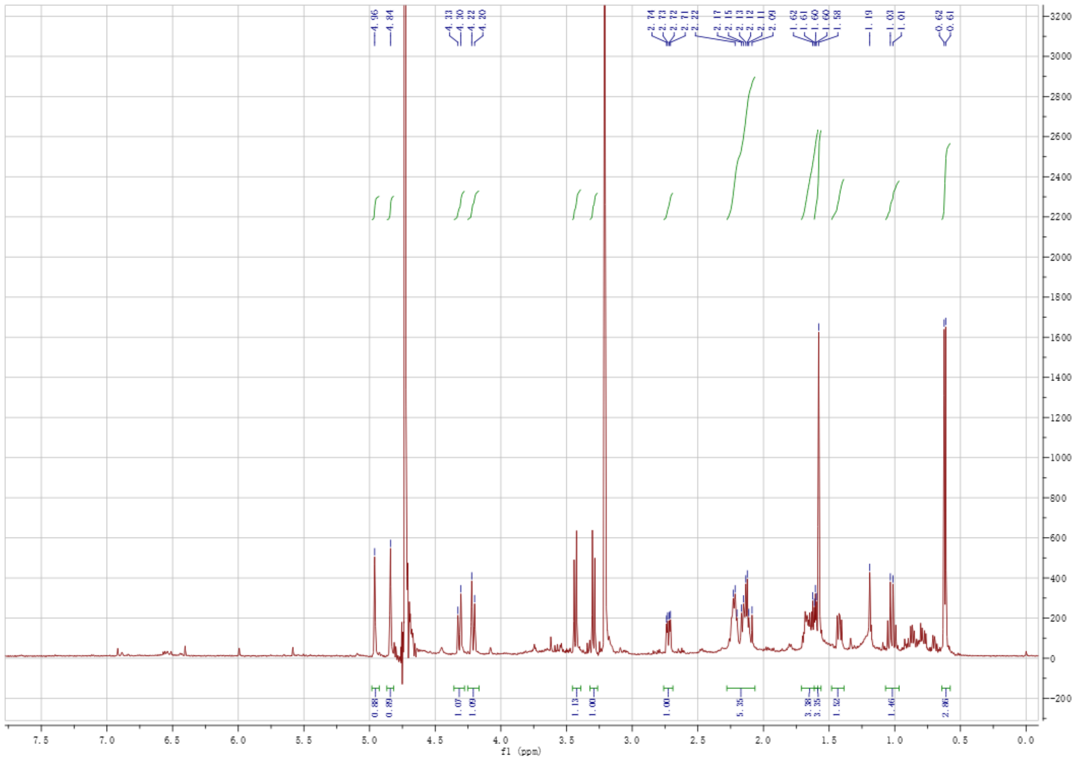

Figure S3. ^{13}C-NMR spectrum of **1**.

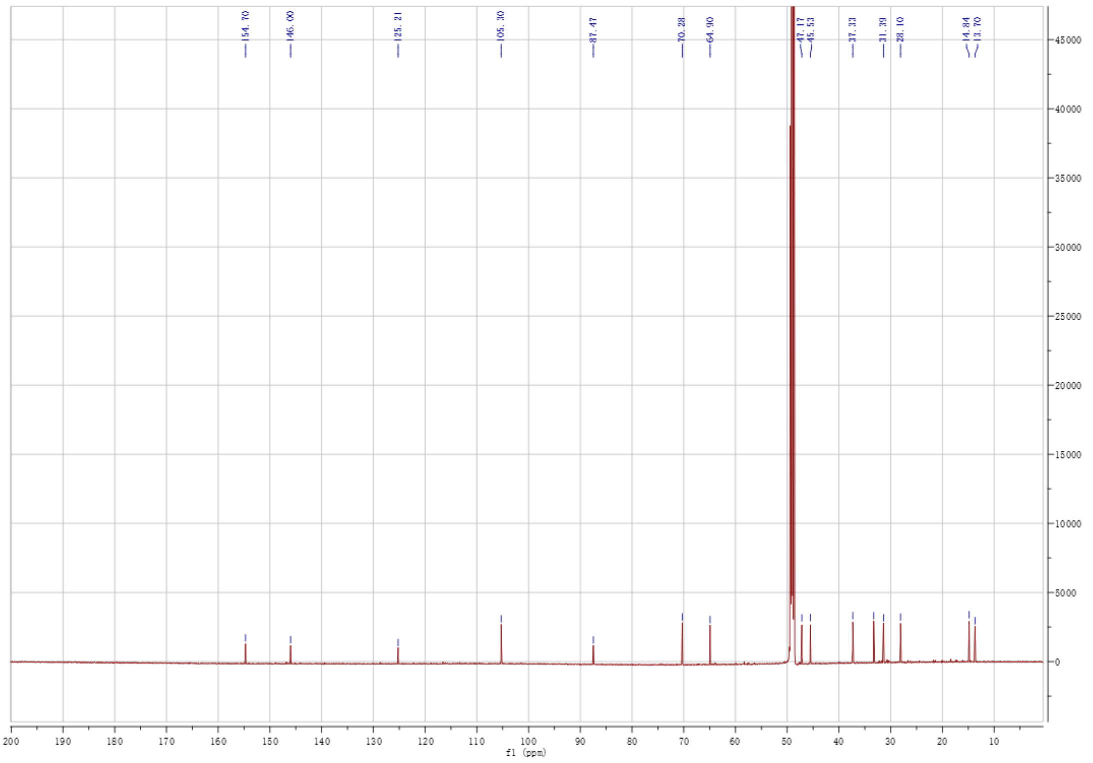

Figure S4. HSQC spectrum of **1**.

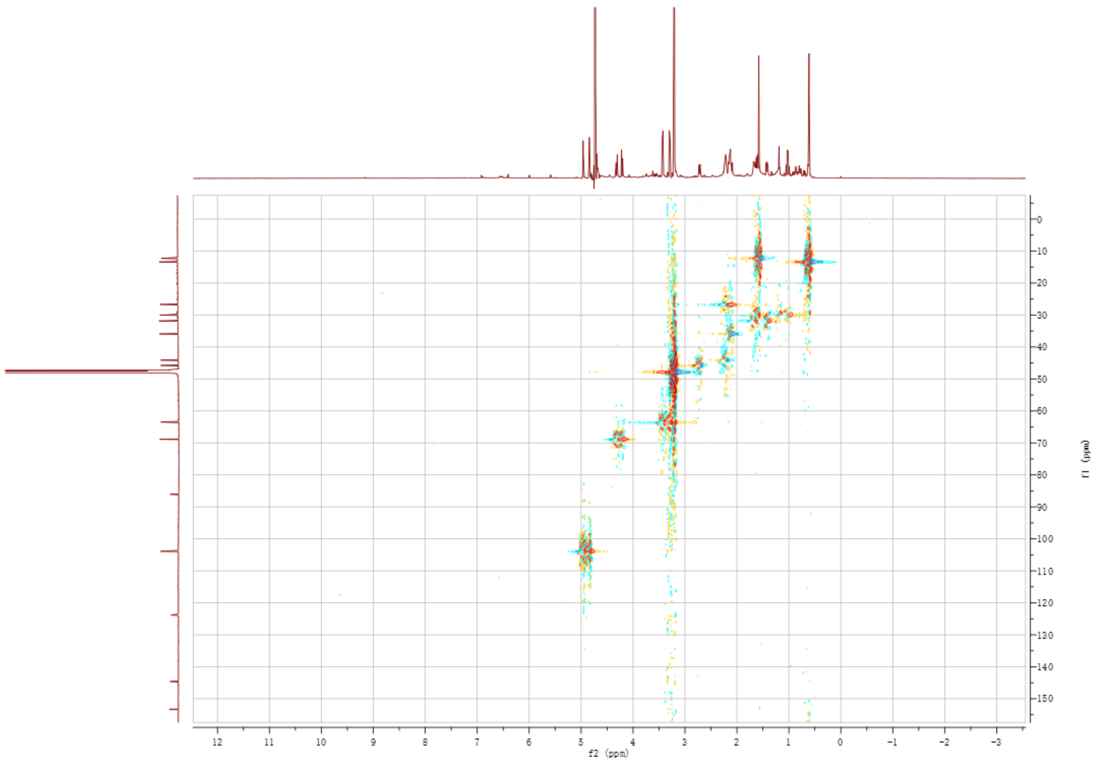

Figure S5. ^{1}H-^{1}H COSY spectrum of **1**.

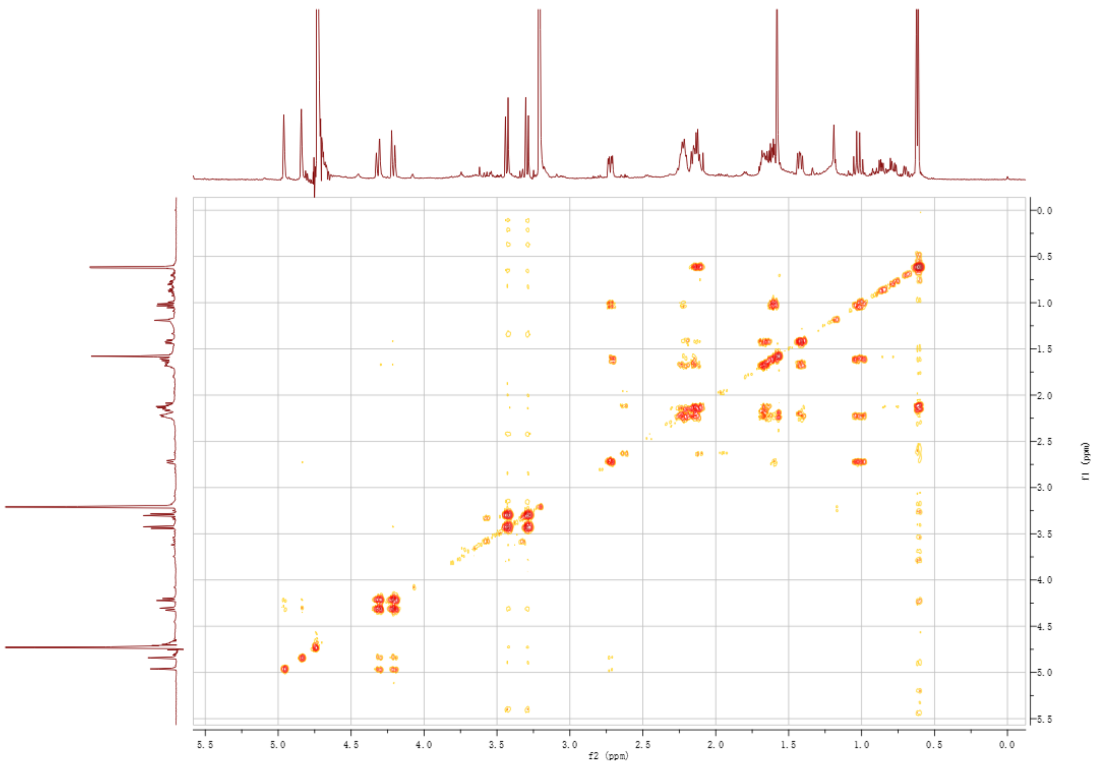

Figure S6. HMBC spectrum of **1**.

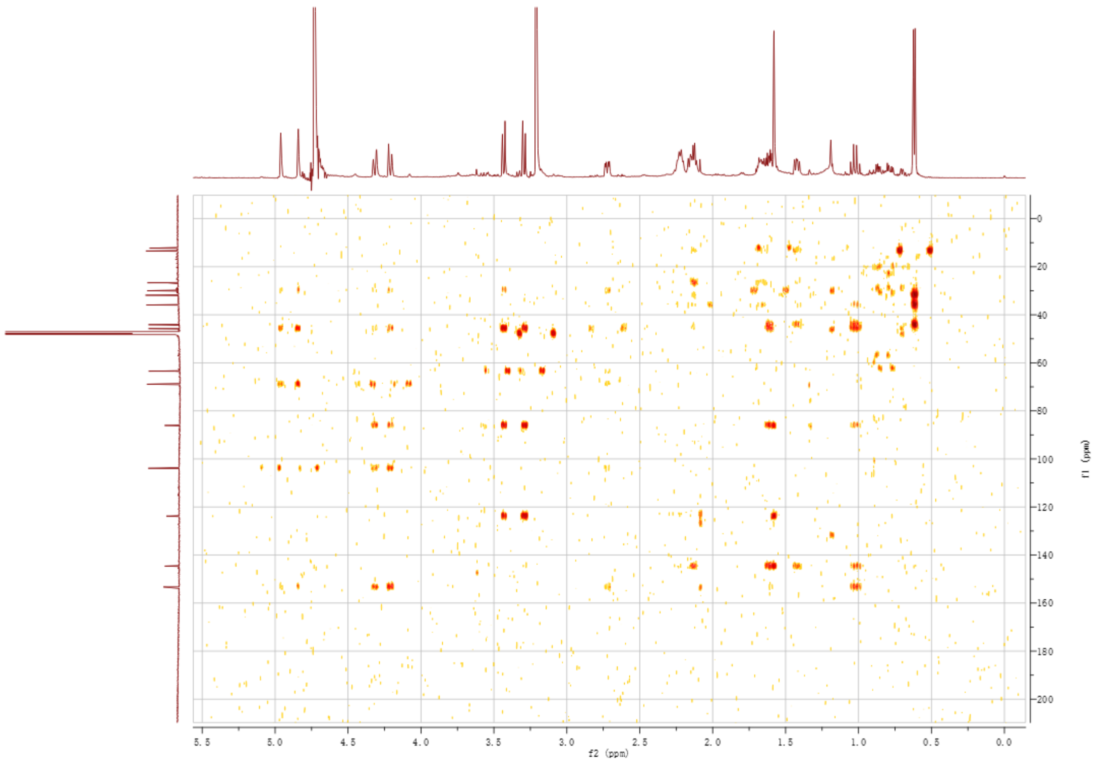

Figure S7. ROESY spectrum of **1**.

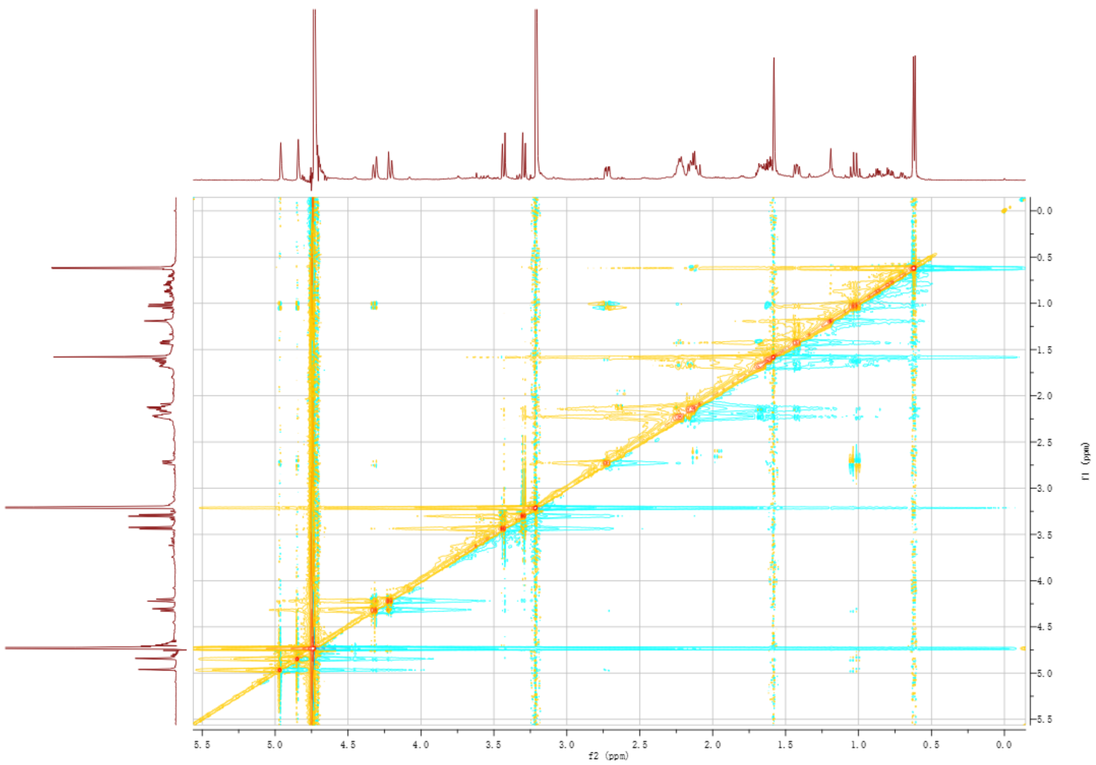

Figure S8. ^{1}H-NMR spectrum of **2**.

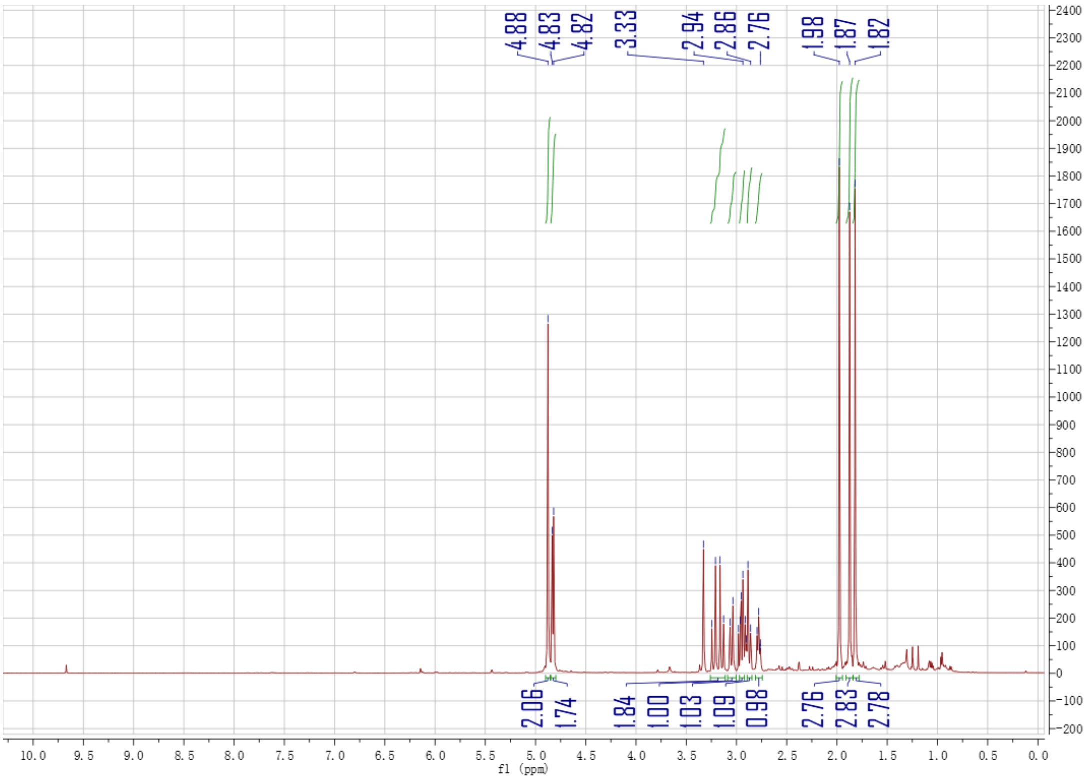

Figure S9. ^{13}C-NMR spectrum of **2**.

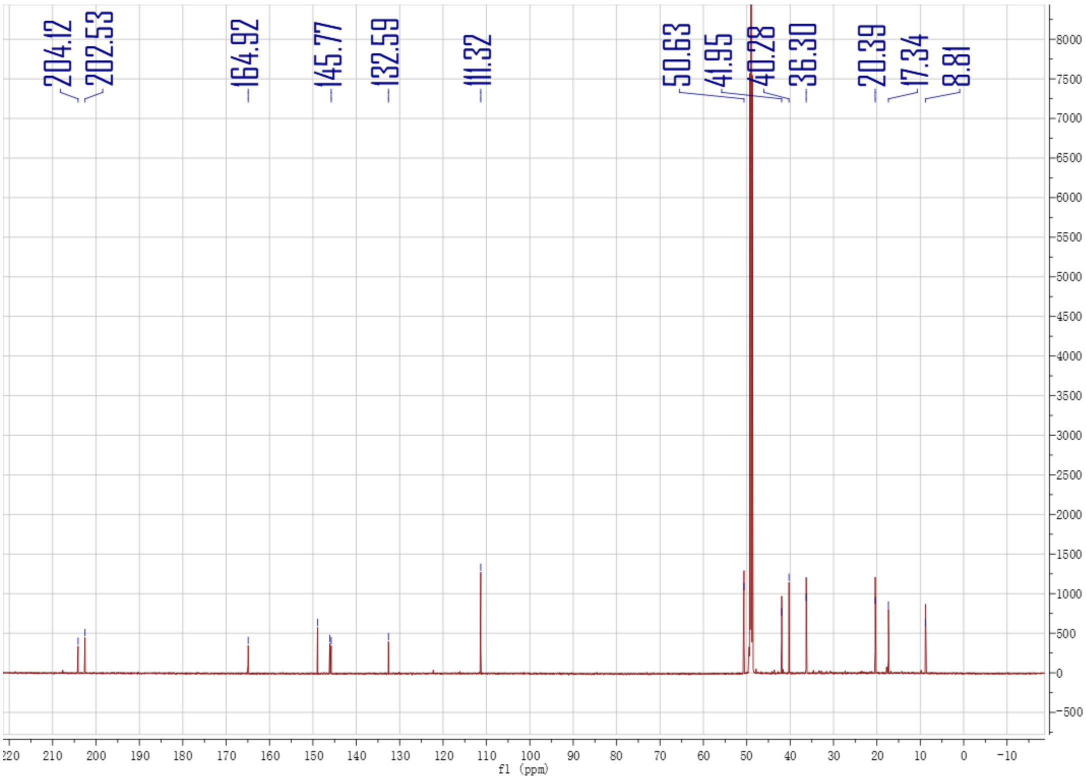

Figure S10. ^{1}H-NMR spectrum of **3**.

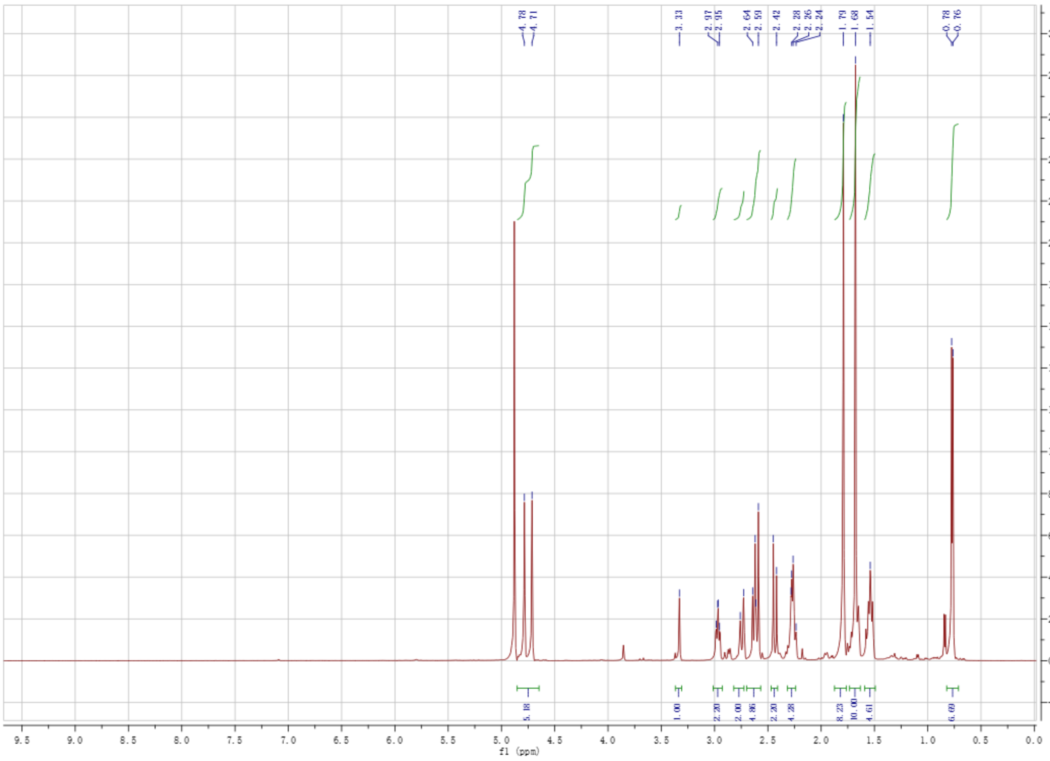

Figure S11. ^{13}C-NMR spectrum of **3**.

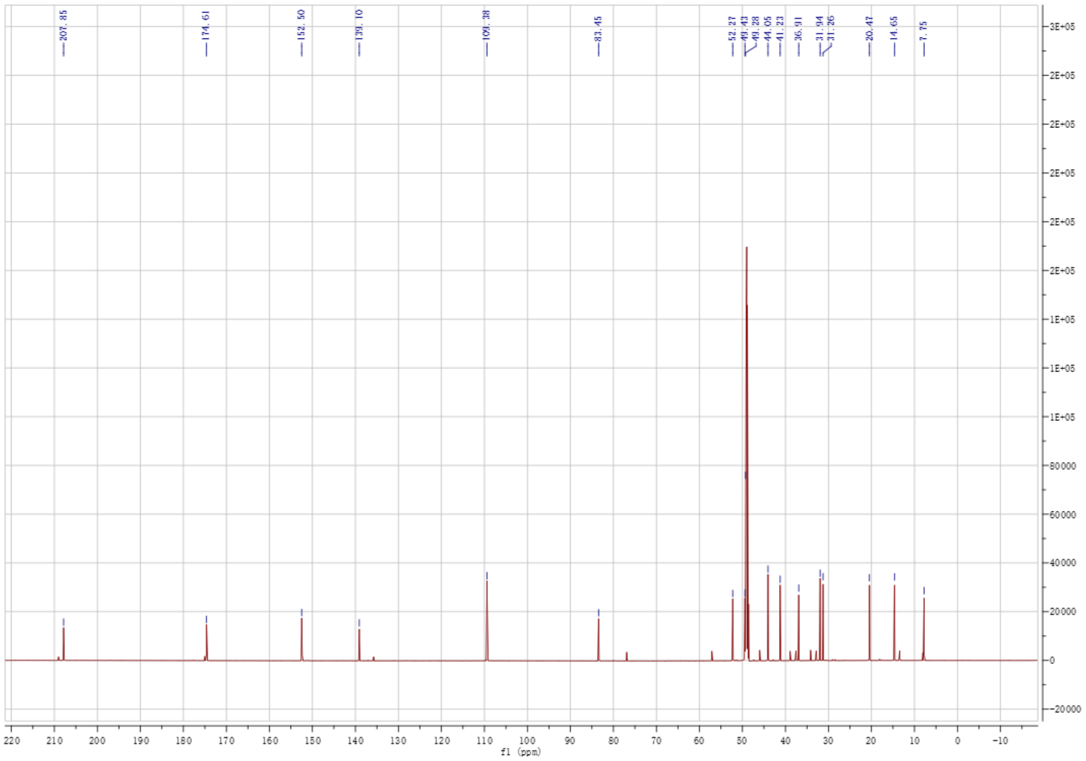

Figure S12. ^{1}H-NMR spectrum of **4**.

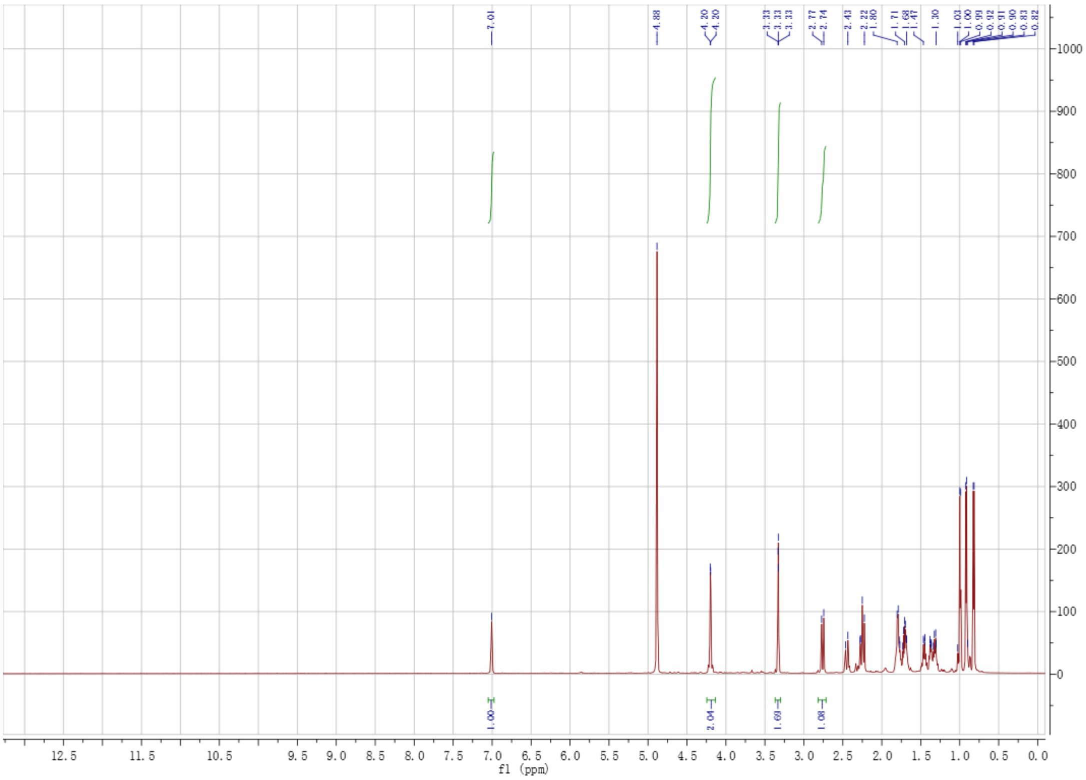

Figure S13. ^{13}C-NMR spectrum of **4**.

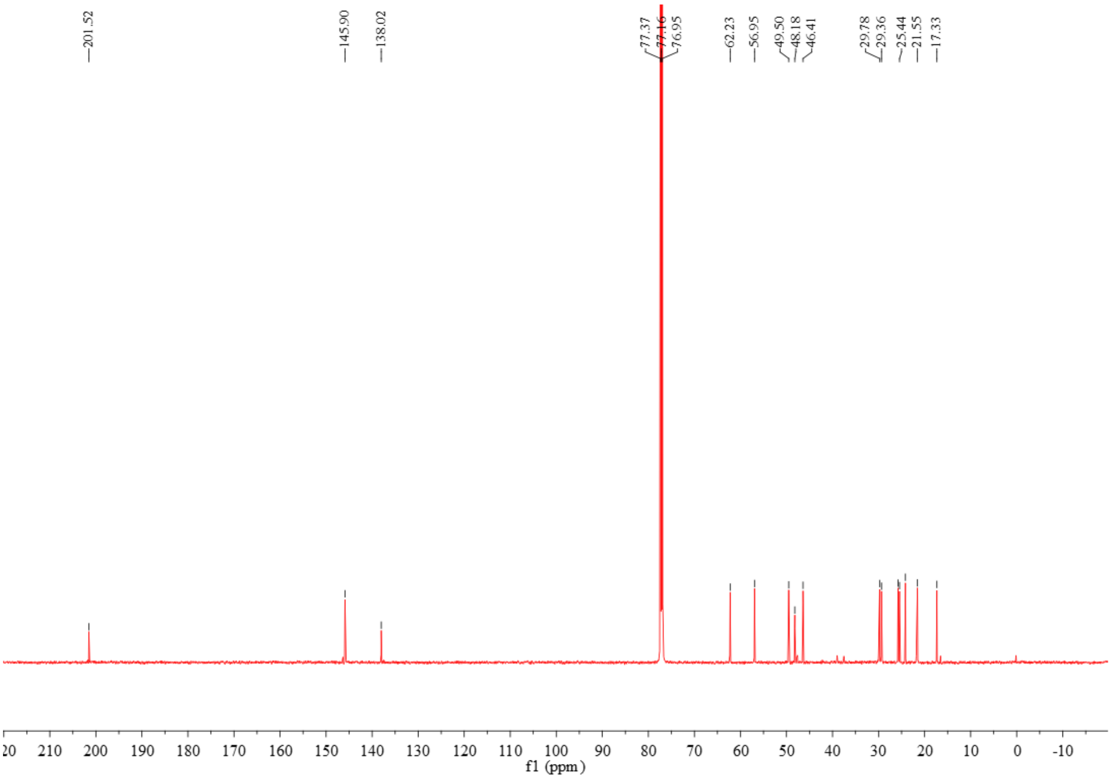

Supplement: Supplementary file 1 [file ijms-24-07357-s001.zip › Supporting Information.pdf]
